# Training Patient Stakeholders Builds Community Capacity, Enhances Patient Engagement in Research

**Published:** 2020

**Authors:** Hannah Cole McGrew, Lidia Regino, Molly Bleecker, Maria Tellez, Blanca Pedigo, Denisse Guerrero, Virginia Sandoval, Loida Varela, Janet Page-Reeves

**Affiliations:** dual specialty nurse-midwife and family nurse practitioner student at the University of New Mexico.; program operations director in the Office for Community Health at the University of New Mexico and co-principal investigator for this project.; senior research scientist 1 in the Office for Community Health at the University of New Mexico. She was data manager for this project.; patient engagement and research coordinator at One Hope Centro de Vida Health Center in Tulancingo Hidalgo, Mexico.; project site director for One Hope Centro de Vida Health Center.; data collectors for this project; data collectors for this project; data collectors for this project; associate professor in the Department of Family & Community Medicine at the University of New Mexico.

## Abstract

Our philosophical framework for research with low-income Latino patients with diabetes prioritizes hiring research staff who share the culture and language of the population of study. Inclusive research design requires an active role by patient stakeholders with training opportunities in a collaborative learning environment to allow patient stakeholder data collectors (PSDCs) to build on existing strengths and expertise. To develop this manuscript, our team reflected on our collective experiences in implementing research-specific trainings for PSDCs. Although our population of study is known to be difficult to recruit and retain, our PSDCs have successfully enrolled participants on schedule, and attrition is low. Although language, institutional requirements, and funding restrictions presented training challenges, we overcame these by using a flexible approach and by incorporating the data collectors’ expertise in refining our protocols. We propose that our success in recruiting and retaining participants is a reflection of our engaged research strategy and framework and demonstrates that engagement promotes better science. However, our experience also demonstrates research institutions need to make policy and infrastructural improvements to reduce barriers and make engaged approaches more feasible.

All group writing and editing sessions used to create this manuscript were conducted in Spanish, the common language among our research team. Patient stakeholder data collector quotes in this manuscript are all translated from Spanish.

Hannah Cole McGrew

## Introduction

A key strategy in patient engaged research is the inclusion of members of the community of study in the development and implementation of research protocols and to incorporate, “their expertise to enhance understanding of a given phenomenon and to integrate the knowledge gained with action to benefit the community involved” (Israel, Schulz, Parker & Becker 1998, p. 173). Supporting patient and community partners with trainings and education to be able to participate in research is not simply acknowledgment of, but a prioritization of community and patient voices, expertise, and authority regarding the topic of study (Hardy, Hughes, Hulen, Figueroa, Evans, & Begay, 2016). The philosophical and ethical framework of engaged research prioritizes hiring individuals from the patient population or the community of study over candidates who may possess more robust formal education or technical skills (Page-Reeves & Regino, 2018; Page-Reeves, Regio, Tellez, Pedigo, & Perez, 2018). However, within the academic paradigm of health sciences research, designing meaningful roles for nonacademic partners to be involved as members of the research team and developing institutional infrastructure to provide education and training are an ongoing challenge. Hiring and training protocols, educational curricula and approaches, and project materials and manuals that are appropriate for use by nonacademic partners are important antecedents for conducting engaged research (Page-Reeves & Regino, 2018; Cené, Haymore, Enga, Sallah, Ritchwood, Wynn, Ellis, & Corbie-Smith, 2015). Through the conduct of a large patient-engaged study, we have identified a bundle of barriers that have created roadblocks for us, but that we believe, given institutional will, could be easily overcome. Yet, these dimensions of research practice are often underappreciated and underreported in the literature.

## Theoretical Background

Previously, among mainstream health investigators, funders, and journals, involving patients in the conduct of research was generally regarded as outside-the-box if not radical. Reviewers for health research funding or for well-known health research journals tended to see patient engagement as antithetical to scientific rigor or to dismiss the engagement component of a project as tangential to the real point of the research. Proposals for research funding and manuscript submissions were inevitably evaluated using a positivistic lens emphasizing quantitative, statistical analyses of outcomes and prioritizing researcher-driven perspectives and objectives. As a result, projects that proposed a patient-engaged research design were rarely funded, and publishing results of an engaged study in high-impact health research journals remained challenging. However, over the past decade, one could argue that patient engagement in research has now become in some senses mainstream^1^ (Wallerstein, Duran, Oetzel, & Minkler, 2017). An engaged project design has been demonstrated to benefit both patients and researchers, and contrary to earlier thinking, to increase the scientific rigor of findings (Balazs & Morello-Frosch, 2013; Haywood, Brett, Salek, Marlett, Penman, Shklarov, Norris, Santana, & Stanizewska, 2015). As patient engagement has become more accepted, funders and universities have jumped on the engagement bandwagon. Yet, despite assertions of institutional support for or even requirements by funders to include engagement in the design of research, there continues to be a lack of infrastructure to allow engagement in health research to actually happen, on the ground.

In this manuscript, we describe our experience training patient stakeholder data collectors (PSDCs) hired from the population of study for a project comparing the cultural competence of two models for diabetes self-management programming for Latinos from low-income households. To prepare this manuscript, we engaged in ex post facto reflection on our methods for hiring PSDCs, the training objectives outlined in our research protocol, and challenges to the practical implementation of appropriate and necessary trainings. All four of the PSDCs on this research project are co-authors on this manuscript. They participated in the preparation of this description of our experiences and revised the final draft, which was translated into Spanish and workshopped as a group.

## Background and Partnerships

We received funding from the Patient Centered Outcomes Research Institute (PCORI) in 2016 to conduct a three-year mixed-method, patient-engaged, longitudinal study (Page-Reeves, Regino, Murray-Krezan, Bleecker, Erhardt, Burge, Bearer, & Mishra, 2017). Our internal shorthand for this project is “PDP,” which stands for PCORI Diabetes Project. The PDP was developed through a collaborative, iterative process with partners and stakeholders from the University of New Mexico (UNM) and One Hope Centro de Vida Health Center, a community clinic operated by East Central Ministries, a faith-based, non-profit that serves a primarily low-income Latino population.

PSDCs were hired and received training during the first three months of the project prior to the beginning of recruitment, which began in February 2017. Our research design anticipated the recruitment and retention of 452 participants (226 pairs of a patient and a corresponding social support). Over a 12-month period, each participant attends four individual appointments with a PSDC (baseline and 3, 6, and 12 months), totaling 1,808 total data collection appointments. During each appointment, the PSDC administers an oral survey. For appointments with the patient participants, the PSDC also gathers biological samples—blood for A1c (diabetes) analysis, height and weight for Body Mass Index (BMI), and a hair sample to test for levels of cortisol as a biomarker for chronic stress. This design requires a significant level of training.

### Hiring PSDCs

The hiring process for the PDP was led by our community partners at One Hope who identified candidates from the community who they knew to be trustworthy and capable. Our research framework prioritized hiring people who spoke Spanish as their first language—either bilingual in Spanish and English, or monolingual Spanish speakers. They also needed to be “patient stakeholders” with personal or professional experience with diabetes—those who were diabetes patients themselves, had family members with diabetes, or who had worked with organizations that support individuals with diabetes from the low-income Latino community (Choi, Heo, Song & Han 2016; George, Duran, & Norris, 2014; Gillis, Lee, Gutierrez, Taylor, Beyene, Neuhaus, & Murrell, 2001; Hardy et al., 2016; McMurdo, Roberts, Parker, Wyatt, May, Goodman, Jackson, Gladman, O’Mahony, Ali, Dicksonson, Edison, & Dyer, 2011; Lloyd Michener, Cook, Ahmed, Yonas, Coyne-Beasley, & Aguilar-Gaxiola, 2012). Other considerations included an understanding of cultural nuances and influence of sociopolitical factors on the health and wellness in the population of study, a sense of commitment and social responsibility to the community, and proven ability to problem-solve and troubleshoot in a low-resource environment.

We knew that it would not be feasible to find candidates who met these requirements and possessed previous experience with research or technical skills outlined in our data collection protocols. In other words, we prioritized experiential wisdom over academic titles or research prowess (Page-Reeves, et al., 2018). We hired individuals who were already affiliated with One Hope in other capacities and therefore, were known to have the qualities we sought.

### Objectives

We created our trainings to enhance the capabilities of PSDCs in the context of our research and promote a bi-directional flow of knowledge, with sessions structured to capture and incorporate expertise from both university and community-based research team members and refine our patient- and community-centered research design. Our PSDCs already possessed the nuanced social and cultural competencies needed to recruit and develop rapport with research participants (Choi et al., 2016; George et al., 2014; Gillis et al., 2001; McMurdo et al., 2011), so we developed and implemented trainings intended to leverage those skills.

PSDCs needed to complete mandatory institutional human research protection and conflict of interest (COI) trainings. UNM utilizes the online Collaborative Institutional Training Initiative (CITI) Group 2 Social & Behavioral Research Investigators modules (citiprogram.org; see https://about.citiprogram.org/en/homepage/) and a university-sponsored online COI training required for all UNM investigators and team members. In addition, PSDCs needed competence for procedures laid out in our research protocol, including:

- Recruiting and consenting participants
- Administering oral surveys (validated surveys and questions developed by the research team) using an iPad and entering data into the Research Electronic Data Capture (REDCap) (Harris, Taylor, Thielke, Payne, Gonzalez, & Cond, 2009) web-based application via the mobile app
- Collecting blood and hair samples
- Accurately measuring height and weight
- Implementing and adhering to protocols for participant scheduling and follow-up

### Training

We conducted 40 hours of PSDC training during the first six months of the project, plus more than 30 hours of ongoing training and support. It was necessary to hold certain trainings at UNM to allow access to specialized facilities, technology, or personnel with technical skills, such as phlebotomists from the UNM Clinical and Translational Science Center (CTSC). However, trainings that were less resource intensive in terms of technology or specialized facilities were conducted at One Hope. The training environment was collaborative and generally informal, with opportunities for team building and shared potluck-style meals. We conducted all trainings and meetings that included the PSDCs in Spanish, and we developed bilingual educational materials for their use. For trainings that required the use of online curricula available only in English or that involved expertise from individuals who did not speak Spanish, bilingual members of the Research Team provided interpretation.

### Mandatory Trainings and Institutional Access

We encountered multiple challenges related to mandatory trainings and obtaining user credentials for affiliate access to UNM electronic systems that would be required for data collection. Trainings required by universities are time-consuming, use jargon and technical language, and often do not pertain to the activities of community-based research staff (Cené et al., 2015). The PSDCs indicated that they felt as if the overarching concepts that were presented in the CITI training were helpful, and that the reasoning behind human protections was valuable to know, but that it was too much information. They suggested that it would have been more useful to do the training in a group setting that fostered discussion about research ethics and how they should be applied to our specific activities. Moreover, neither the CITI nor the COI training were available to us in Spanish. Although the CITI training does exist in a Spanish version, the Spanish training was not supported at UNM. As a result, both trainings had to be completed by our non-bilingual PSDCs with the assistance of interpretation. This made the already lengthy and demanding experience of taking the training even more tedious, averaging six hours for the bilingual PSDCs, and nine for non-bilingual PSDCs. This was a cumbersome process and we advocate for the availability of trainings in languages other than English. However, the trainings conducted with interpretation did provide an unanticipated opportunity for relationship building through the interaction that was required between university and community team members.

Similar bureaucratic barriers exist in relation to obtaining access to institutional systems for the secure transfer of participant data and protected health information. While UNM has channels for affiliates to complete mandatory trainings without credentialed access to UNM systems, credentials are needed for the virtual private network (VPN), the REDCap database, and the REDCap app that we planned to use for data collection. It is challenging for people who are not UNM employees to gain access to these systems because of a multi-step process that requires the creation of several password protected accounts and layers of internal approvals that are not uniform for all types of affiliates. Not only was the process convoluted and sometimes contradictory, portions of the training could not begin until all PSDCs had been approved for access, meaning that our entire research enterprise was held up for indeterminate periods of time. From the perspective of the PSDCs, this process and the management of these new accounts—each with different steps for access—was unnecessarily frustrating. It was clear to us from this experience that university infrastructure needs to be honed and streamlined to better support engaged research.

### Phlebotomy

We draw blood at half of our data collection appointments, so PSDCs need to be skilled phlebotomists. Because we prioritized hiring patient stakeholders, we had to provide this training. We started the phlebotomy training concurrently with the mandatory trainings at the outset of the project. Phlebotomy was the most challenging training to coordinate since funder guidelines had disallowed inclusion of phlebotomy training costs in our budget. Initially, we were nearly stumped in our attempts to find a way to train non-academic, and non-university affiliated staff in phlebotomy. Elsewhere (Page-Reeves, Regino, McGrew, Tellez, Pedigo, Overby, Cunningham, Tiggert, & Burge, 2018), we chronicle the infrastructural challenges we faced and how through outside-the-box thinking and collaboration, we partnered with the UNM CTSC lab to use protocols they follow for training their own lab staff to develop and conduct the training for our PSDCs.

The phlebotomy training consisted of a half-day intensive orientation with follow-up sessions each week for five weeks to practice venipuncture technique under the supervision of a certified phlebotomist. Our team provided interpretation. Although we had planned to use headsets for simultaneous interpretation, the equipment malfunctioned and we had to rearrange the training space so that the interpreters could sit behind the non-bilingual PSDCs, providing a mix of simultaneous and consecutive interpretation. This created an unusual communication dynamic and was difficult for the interpreters, especially during periods of discussion or questions.

In addition to language, there were also challenges presented by having a mix of UNM CTSC staff, project research staff, and PSDC trainees who came from diverse backgrounds, both medical and non-medical. Clarification and discussion of terminology was often needed for clear communication. For example, the translation of venipuncture in English, to *venopunción* in Spanish was relatively meaningless to someone unfamiliar with medical terminology. The interpreters translated the word, but then also had to describe how venipuncture referred to the act of inserting a needle into a vein to draw blood. Although this process extended the length of the training, these were important detours that were necessary to ensure that the PSDCs developed phlebotomy competency and that they felt confident in their comprehension of the material, and so that UNM CTSC trainers could effectively address the questions and concerns of the trainees.

The PSDCs report that they were initially intimidated by the idea of having to draw blood. They worried about causing pain for the participant, or that they would be too nervous. However, because of the quality of the training provided by the UNM CTSC and the capacity created through the group process of interpretation and discussion, they overcame their fears and developed confidence in their abilities. One PSDC said, “When I saw those needles I thought ‘Wow! I’m never going to feel comfortable doing this!’ but as it turns out, I’m really good. It’s valuable to me to know that I could overcome that.”

### Recruitment and Consenting

Learning to recruit and consent participants was another core training component. PSDCs practiced using a generic script with language about research activities and participation incentives. In the trainings, we emphasized the most important aspects of the script while the PSDCs provided insight into how they felt potential participants would respond. The PSDCs developed strategies for ensuring that participants with no experience participating in research were fully informed about the study and what their participation would entail. We created detailed operational protocols for these processes, and the PSDCs practiced through role play with members of the Research Team and with each other.

### Oral Survey

Administering the oral survey in our project requires the PSDCs to be familiar with using an iPad and with the REDCap App. To give them these skills, we mixed hands-on practice with peer-learning. This approach accommodated differences in PSDC technological knowledge and capability while leveraging individual strengths. Elsewhere we have discussed the challenges we faced in developing a database appropriate for use by PSDCs (Bleecker, McGrew, Regino, Erhardt, Mishra, Bearer, Tellez, Wesley, & Page-Reeves, n.d.). We created our REDCap database to be dual language, with both English and Spanish translations for all questions and instructional text, and we utilized the REDCap App Spanish translation interface. However, certain warnings, alerts, and hyperlinks were “fixed” in English, so we developed detailed instructions in our manual of operations and added visual materials with instructions for what to do.

In the process of learning to administer the oral survey, the PSDCs actively participated in the design and revision of the survey format. The oral survey consists primarily of validated surveys and we were not able to modify the content in most cases. However, while it was important for the PSDCs to understand methods and protocols for accurate and rigorous data collection, we were not teaching them to deliver an oral survey by merely reading questions off their iPads. Rather, we worked with them to tap into the skills they possessed through role-playing, with the PSDCs practicing administration of the survey to each other. The PSDCs identified concerns about challenging or problematic language, and they educated the university Research Team members about portions of the survey they thought would solicit mixed or adverse responses from participants. Through this co-learning process, we have been able to anticipate problems and to collaboratively develop strategies to address those issues in a sensitive, patient- and community-centered way.

The PSDCs saw these role-plays and collaborative sessions as the most valuable part of the training. It was a safe environment to put their skills into practice and receive feedback or advice from other members of the team. One PSDC described this as, “We took all of the tools and information, and then we made them our own.”

### Additional Trainings

Since we began data collection in February 2017, unanticipated issues have arisen and we have worked collaboratively with the PSDCs to develop solutions and make appropriate modifications to the protocols in our operations manual. We have addressed unforeseen technical challenges related to use of the iPads and REDCap, and participants unexpectedly revealing that they are experiencing behavioral health or domestic violence crises during data collection appointments. We developed follow-up trainings and invited community experts to share their wisdom on these topics. The PSDCs say that, while they felt the trainings empowered them to confidently perform the research activities, the ongoing support is the most valuable. One PSDC reported that “no matter how prepared you think you are, issues always come up. So, these follow-up sessions are important. We feel like we can call [on the team] any time and [they] hear us… . We meet to talk about ways to do it better, and then we practice.”

### Preliminary Outcomes

Despite challenges that emerged in the process of hiring and training PSDCs, we hired four highly competent individuals who each completed all trainings and learned to operate effectively as data collectors for this project. Even though our population of study is known to be difficult to recruit and retain, at this preliminary stage in the project, our PSDCs have successfully recruited and enrolled 452 participants (226 patient-social support pairs) on schedule, which was challenging given that it is a hard-to-reach population, and we are finding that attrition is incredibly low, which is also notable given that this population tends to have a high attrition rate. In the final nine months of the three-year study, our attrition for patients (upon which the study is based) is seven of 226 or 3.1%. Moreover, information that the data collectors are gathering has been consistent and accurate, with minimal missing data. In fact, the data collectors identified problems with branching logic in the design of the database that was resulting in missed data, and they have been flexible and accommodating of changes to the survey tools to address these.

## Discussion

The meaningful inclusion of patient stakeholders is fundamental to developing equitable health research projects. They contribute expertise that makes the research rich and insightful. Hiring and training frameworks for engaged research should be built around capable members of the community, and not the other way around. The rapport the PSDCs have developed with participants extends beyond the scope of the research. Participants have reported that when they attend data collection appointments, they feel like the PSDCs are looking out for them. This is not surprising when you consider a comment by one PSDC:

People deserve to be listened to. Even if they aren’t talking about the survey—if they want to tell me about their mother, or their sister who’s sick, or their son that died, I am not going to shut them up and move on. I am going to get the work done, but I am going to hear them.

This attitude is central to the ethic of our research and demonstrates the PSDCs’ personal stake in and commitment to the community. The PSDCs agree that this experience has given them new skills that will help them in their careers.

Moreover, hiring PSDCs leads to good science. In the process of learning to administer the oral survey, the PSDCs actively participated in improving the design of the survey, the survey content, and methods for administration. Also, involving PSDCs in the way that we have done not only impacted the PSDCs themselves, but also the dynamics of the research team and the attitudes of team members not previously experienced in community-engaged research. This impact therefore will continue to reverberate.

### Lessons Learned

On the one hand, we learned positive dimensions of the engagement process. PSDCs demonstrated that they are highly adaptable and that they have the capacity to be responsive to obstacles they confront on the job. On the other hand, we also became more fully cognizant of the barriers to engaged research through the hurdles that we faced in hiring and training PSDCs. These included language issues, inflexible institutional environments, and lack of infrastructure to support engaged research rather than a lack of ability on the part of the data collectors themselves. To surmount enormous challenges, we were confronted with the constant need for creative work-arounds that were not efficient or cost effective. There were institutional processes that were not streamlines and not clearly documented or defined. Not only does this not align with industry best practice, but it requires persistence beyond all reason. There were so many places where we easily could have thrown our arms in the air and given up. The result was a huge expense in terms of staff time that substantially took away from content-related research activities and caused frustration on the part of staff and affiliate partners.

### Dialogue

In our process of working on solutions, we have discovered that you cannot take no for an answer. Engaging in dialogue with university leadership can result in policy change to remove infrastructural barriers to engaged research. Some of the issues we identified have been resolved; some we are continuing to work on, some we developed feasible work-arounds, and some we were told no, but we are still working on them, if indirectly.

### Infrastructure and Policy Environment

It is projects like ours that are challenging universities to create infrastructure to support community- and patient-engaged research by adopting policies and providing clear and accessible processes. As health research that includes meaningful engagement and participation of patients and community members moves toward the mainstream, academic institutions and funders will have to reconsider existing strategies, policies, and infrastructure to support bringing non-university, community affiliates into the fold (Cené et al., 2015; Lloyd Michener et al., 2012). Making these changes will require collaboration and innovation. While some investment in time and energy may be needed to bring patient stakeholders up to speed in terms of specific technical skills or institutionalized trainings, we are seeing that the benefit to the research and to the community is more than worth it. University responsiveness will be key in continuing to build and improve infrastructure for engaged research in the future.

### Transformation

Our experience demonstrates the potential transformative effects of an engaged research design. PSDC experienced transformative personal growth. Participants experienced a new, more meaningful interface with a research study. Our research results reflect positive impacts related to recruitment, retention, and design. And other research team members who were not formerly inclined to engage in engaged research have begun to see its value. We clearly show that engagement and science can be integrated, successful, and powerful.

## Footnotes

1 For example, in the United States: National Institutes of Health (NIH) (PA-13-209) Innovative Measurements Tools for Community Engaged Research Efforts; National Institutes of Health/ National Institute of Nursing Research (PA-14-142) Community Partnerships to Advance Research; American Cancer Society Midwest Division (CDC)-RFA-PS-14-1406) Pilot and Exploratory Studies Using Community-Based Participatory Research (CBPR) to Achieve Cancer Health Equity; and Center for Disease Control (CDC) (CDC-RFA-PS 14-1406) Community Approaches to Reducing Sexually Transmitted Disease. And in the United Kingdom: the Clinical Research & Innovation Office Lay Advisory Panels and PPI team; National Institute for Health Research (NIHR) Research Design Service for Yorkshire & the Humber (RDS YH) funding awards; NHS Involve; and People in Research, Oxford Clinical Trials Research Unit.
